# Plaques Do Not Act Alone: Time to Redefine Coronary Vulnerability from Lesion to Phenotype

**DOI:** 10.3390/jcm14217568

**Published:** 2025-10-25

**Authors:** Sara Sgreva, Sara Essa Alsubai, Emiliano Bianchini, Foziyah Alqahtani, Paolo Alberto Del Sole, Hesham Elzomor, Ruth Sharif, Simone Fezzi, Faisal Sharif

**Affiliations:** 1 Department of Cardiology, Galway University Hospital, Newcastle Rd, H91 YR71 Galway, Ireland; 2 Sharif Cardiovascular Research Group, University of Galway, H91 YR71 Galway, Ireland; 3 Research Ireland Centre for Medical Devices, University of Galway, H91 TK33 Galway, Ireland; 4 Division of Cardiology, Department of Medicine, Verona University Hospital, Piazzale A. Stefani 1, 37126 Verona, Italy; 5 School of Medicine, University of Galway, H91 TK33 Galway, Ireland

**Keywords:** coronary artery disease, vulnerable plaque, CCTA, radial wall strain

## Abstract

The classical concept of plaque vulnerability, centred on specific morphological features, has failed to deliver reliable risk prediction in clinical practice. Recent evidence highlights the need to redefine coronary vulnerability as a dynamic, patient-specific phenotype shaped by plaque biology, systemic inflammation, and haemodynamic forces. Advanced imaging modalities, artificial intelligence, and circulating biomarkers now enable a multidimensional assessment of this complex phenotype. This integrative approach may offer a more precise framework for risk stratification and personalised prevention in coronary artery disease.

## 1. Introduction

Coronary artery disease (CAD) remains a leading cause of morbidity and mortality worldwide, and despite advances in prevention and therapy, acute coronary syndromes (ACS) continue to represent a significant healthcare burden [1]. Most ACS events arise from the destabilisation of so-called vulnerable plaques, yet predicting which lesions or patients are truly at risk remains a major clinical challenge—both in primary and secondary prevention [2]. Even after successful revascularisation and optimal medical therapy, a residual risk of recurrent coronary events persists [3]. Historically, risk stratification has relied on the assumption that myocardial ischaemia reflects severe stenosis and high event risk; however, this model fails to capture the full complexity of coronary and patient vulnerability [3,4]. The Providing Regional Observations to Study Predictors of Events in the Coronary Tree (PROSPECT) study supported this by showing that many non-culprit plaques leading to events appeared angiographically mild at baseline: intravascular ultrasound (IVUS) identified key features such as thin-cap fibroatheroma (TCFA), large plaque burden, and small luminal area as strong predictors of major adverse cardiac events (MACE), highlighting the limitations of angiography alone [4]. The “mild” appearance of the stenosis was largely due to positive remodelling, a hallmark of metabolically active, inflamed plaques that evade detection with conventional angiography [5].

Nonetheless, plaque morphology alone is insufficient to reliably predict events. Plaque rupture and healing occur along a continuum, shaped by dynamic biological processes involving inflammation, lipid metabolism, and immune processes that an image cannot fully capture [6,7]. This may explain the modest prognostic value of high-risk features observed in trials such as Scottish COmputed Tomography of the HEART Trial (SCOT-HEART), where only 4.1% of 608 patients with high-risk plaque features on coronary computed tomography angiography (CCTA) experienced myocardial infarction (MI) or coronary death over 4.7 years [8]. Similarly, in the PROspective Multicentre Imaging Study for Evaluation of Chest Pain (PROMISE) Trial, only 4.8% of 505 patients with non-obstructive CAD but high-risk plaques on CCTA had cardiac events over two years [9].

**Figure 1:**
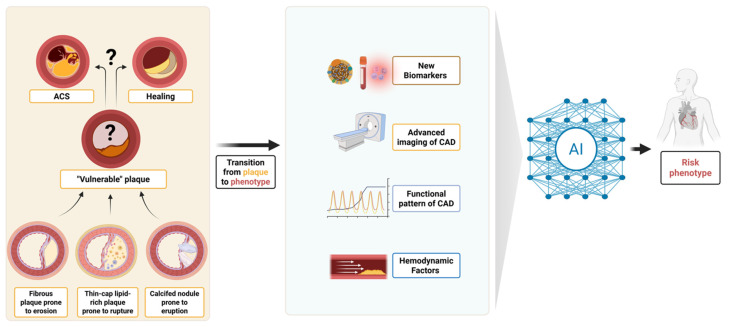
**(Central Figure) From plaque to phenotype: redefining coronary vulnerability.** Evolution from the traditional lesion-centric concept of the “vulnerable plaque” towards an integrated “vulnerable phenotype” approach. ACS, acute coronary syndrome; CAD, coronary artery disease; AI, artificial intelligence.

The classical concept of “plaque” has thus proven reductive, overlooking the systemic nature of atherogenesis and the interplay between local lesions and broader patient-specific factors. In this review, we explore how invasive and non-invasive imaging modalities, biological profiling, artificial intelligence (AI), and biomechanical modelling may contribute to shifting from a static, lesion-based view of vulnerability toward a more integrated and dynamic risk phenotype. Rather than focusing solely on plaque morphology, we discuss how emerging multimodal tools can characterise vulnerability as a multidimensional and patient-specific process, Figure 1 (Central Figure).

## 2. Vulnerable Plaque: Varying Definitions and Pathophysiological Mechanisms Leading to ACS

The concept of the vulnerable plaque—first introduced by Müller et al. in the 1980s—refers to an atherosclerotic lesion prone to destabilisation and thrombotic occlusion, often resulting in ACS [10]. This concept emerged from two clinical needs: identifying lesion types likely responsible for ACS and understanding the underlying biological processes to guide targeted therapies [10]. In 2003, Naghavi et al. published the first consensus document to standardise the definition of vulnerable plaques and identified five major criteria: (1) thin fibrous cap with a large lipid core; (2) active inflammation (macrophage infiltration); (3) endothelial denudation promoting platelet aggregation (plaque erosion); (4) plaque fissure indicating recent rupture; and (5) diameter stenosis >90% [11,12].

Despite decades of investigation, these insights have had limited clinical impact, partly due to the retrospective and heterogeneous nature of histopathological studies. Further complicating the ongoing search for a univocal definition, three clearly distinct primary morphological substrates for coronary thrombosis have been identified: plaque rupture (70% of cases), plaque erosion (25%), and eruptive calcified nodules (5%) [13]. These three morphological substrates are associated with specific plaque phenotypes, inflammatory profiles, and mechanisms of destabilisation: plaque rupture typically involves lipid-rich necrotic cores and thin fibrous caps infiltrated by macrophages and activated T cells; in contrast, plaque erosion is characterised by an intact but denuded endothelium, extracellular matrix exposure, and platelet adhesion [14]. Finally, calcified nodules, though rare, protrude into the lumen, disrupting flow and triggering thrombosis via surface irregularities and turbulence [15].

## 3. Multiple Invasive and Non-Invasive Modalities to Assess Plaque Vulnerability

**Figure 2:**
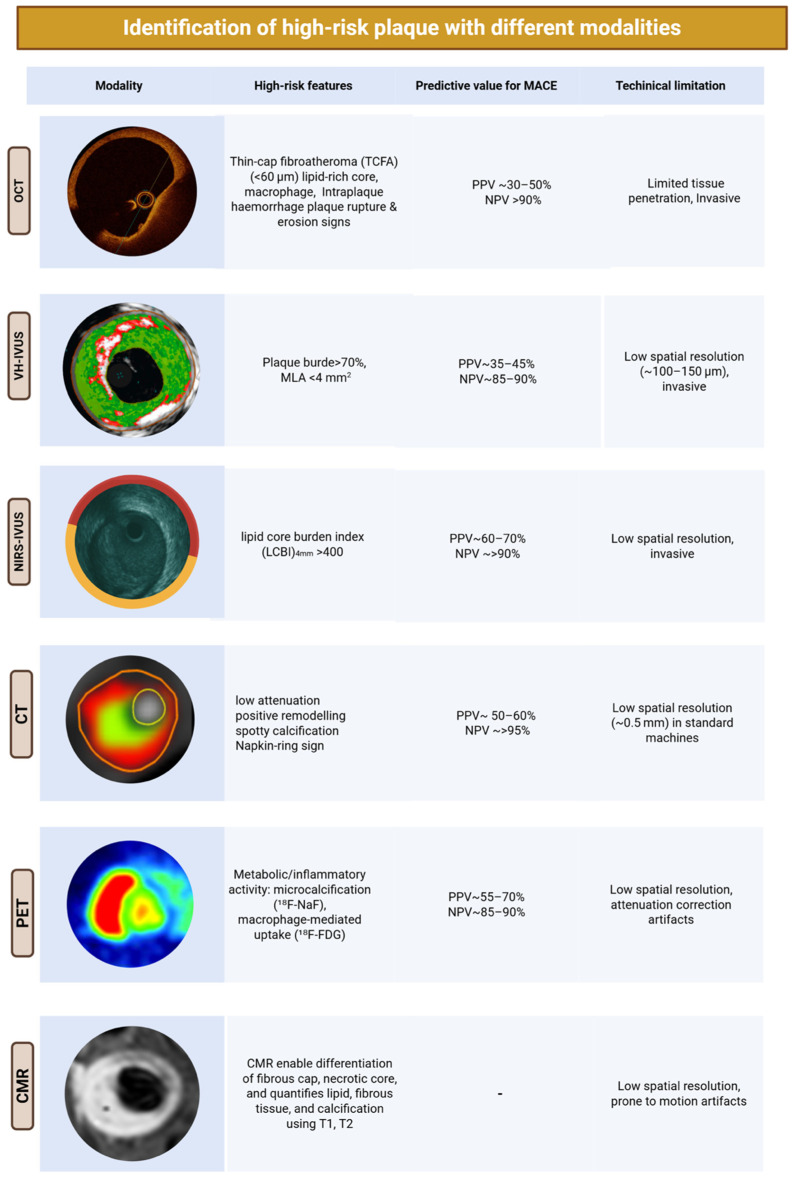
**Imaging toolkit for assessing coronary plaque vulnerability.** Main invasive and non-invasive imaging modalities used to characterise coronary plaque.

Different imaging methods, both invasive and non-invasive, have been developed to characterise plaque morphology and composition, as well as to identify vulnerable features. The differing physics, technologies, and modalities of these imaging tools have led to a wide array of reports. While this approach has helped highlight anatomical characteristics potentially linked to biological processes, it has also tended to oversimplify—and at times confound—their true clinical significance within the broader and more complex context of coronary atherosclerosis, Figure 2.

### 3.1. Invasive Imaging

To overcome the limitations of coronary angiography, several intravascular imaging modalities have been developed to characterise high-risk plaque features in vivo. Among the most widely used in catheterisation laboratories are IVUS, virtual histology IVUS (VH-IVUS), optical coherence tomography (OCT), and near-infrared spectroscopy (NIRS) [16].

- *IVUS* allows visualisation of the arterial wall and lumen using ultrasound waves reflected from the vessel wall. It can identify plaque composition, rupture, and intraluminal thrombus, although its axial resolution (~150 μm) is insufficient for reliably measuring components of vulnerable plaques like the fibrous cap or the presence of macrophages infiltrates [17].
- *VH-IVUS* was initially introduced in 2002 and has been developed in collaboration with the IVUS catheter to overcome IVUS’s limitations. It uses spectral analysis of IVUS radiofrequency signals to generate colour-coded maps of plaque composition: fibrous (dark green), fibro-fatty (light green), necrotic core (red), and dense calcium (white) [18]. Both the ATHEROREMO-IVUS and the PROSPECT study confirmed that VH-IVUS TCFAs were significantly associated with death or ACS at one year (HR 2.56; 95% CI 1.18–5.54; *p* = 0.017) [19,20].
- *OCT* employs near-infrared light to provide high-resolution imaging (10–20 μm) of coronary plaques, allowing detailed assessment of microstructural features such as fibrous cap thickness, lipid pools, macrophage infiltration, neovascularisation, microchannels, plaque ruptures, erosions, and thrombi [21]. This unparalleled resolution makes OCT the imaging modality of choice for identifying TCFA and assessing features associated with plaque vulnerability. However, due to its limited tissue penetration (1–1.5 mm), OCT is less suitable for evaluating total plaque burden and vessel remodelling. Four OCT-derived features have been most consistently associated with plaque vulnerability:
  ○ Fibrous cap thickness < 75 μm, the strongest predictor of rupture and adverse events.
  ○ Minimal lumen area (MLA), which reflects the degree of luminal narrowing but remains a debated threshold. MLA cut-offs considered “high-risk” vary by coronary segment and clinical context: values between 3.5 and 4.5 mm^2^ are frequently reported, but the optimal threshold depends on vessel size, lesion location, and patient-specific factors [22,23,24].
  ○ Lipid arc extension > 180°, representing the circumferential spread of necrotic lipid.
  ○ Intraplaque macrophage infiltration, reflecting local inflammation.
  In the CLIMA study, which focused on non-culprit plaques in the proximal left anterior descending artery (LAD), the simultaneous presence of TCFA < 75 μm, MLA < 3.5 mm^2^, lipid arc > 180°, and macrophages in a single plaque identified lesions at highest risk of future MACE [25].
- *NIRS* enables precise detection of intraplaque lipid content and cholesterol. It is currently the most established catheter-based technique for lipid detection, with Food and Drug Administration (FDA) approval. It also provides insights into inflammation, cell proliferation, and apoptosis. When co-registered with IVUS or OCT, it allows three-dimensional plaque visualisation, enhancing the detection of high-risk lesions [26].

### 3.2. Non-Invasive Imaging

- *CCTA* is the most widely available non-invasive technique for coronary artery imaging. Recent clinical guidelines emphasise the role of CCTA as a first-line examination for evaluating patients with suspected CAD, particularly those with low-to-intermediate “pre-test” likelihood [27]. Moreover, both European and American Guidelines recommend the use of CCTA to rule out ACS in patients presenting with acute chest pain and an inconclusive initial evaluation [28,29]. Advances in CCTA technology and software have enabled accurate evaluation of coronary plaque composition and morphology [30]. Detectable vulnerability features validated in multiple studies were as follows:
  ○ Spotty calcification: calcified foci (≥350 HU) ≤3 mm in diameter, surrounded by non-calcified plaque.
  ○ Low-attenuation plaque: non-calcified plaque with attenuation ≤30 HU, distinct from perivascular fat.
  ○ Positive remodelling: an increase in vessel diameter ≥10% relative to a reference segment (remodelling index > 1.1).
  ○ Napkin-ring sign: a central low-attenuation zone adjacent to the lumen, surrounded by a higher-attenuation rim (<130 HU).
- *Photon-counting computed tomography (PCCT)* represents a newly introduced detector technology in the realm of CCTA scanning. PCCT uses direct conversion detectors—typically based on cadmium telluride or silicon semiconductors—to transform incoming X-ray photons directly into electrical signals. Unlike conventional computed tomography, this enables energy-resolved imaging by categorising photons into discrete energy bins, thereby improving tissue characterisation. PCCT offers higher spatial resolution (pixel size down to 0.15–0.225 mm) and reduced electronic noise, which makes it particularly suited for detailed plaque assessment in atherosclerosis. Emerging evidence supports the diagnostic superiority of PCCT over conventional energy-integrating detector in coronary plaque assessment [31].
- *Cardiac magnetic resonance (CMR),* using different sequences, allows the detection of the TCFA and intraplaque components such as necrotic core, macrophages, haemorrhage, neovascularisation, calcifications, and subclinical plaque rupture; it also permits quantification of lipidic and fibrous tissues [32]. However, given the deep intrathoracic location of coronary vessels, CMR’s sensitivity is lower than that of CCTA. This, along with lower availability and susceptibility to breathing- and cardiac motion-related artefacts, limits its use in the assessment of plaque vulnerability [32].
- *Positron Emission Tomography (PET)* enables in vivo imaging of the metabolic activity of atherosclerotic plaques through radiotracer uptake, offering insight into disease pathophysiology. PET can visualise key biological features to assess distinct phases of inflammation or specific components of plaques [33]. Increased PET activity has been associated with adverse cardiac events [33]. Despite its capability to non-invasively characterise plaque biology, its clinical use remains limited because of background uptake of glucose in metabolically active myocardium, cardiac and respiratory motion, and the lower spatial resolution of PET; moreover, most radiotracers are still investigational and not approved for routine clinical use [33].

## 4. The Limits of the Vulnerable Plaque Concept

Despite significant advances in imaging and pathology, the vulnerable plaque paradigm has had limited impact on clinical practice: identifying plaques that will lead to future events remains an elusive goal. In a recent meta-analysis of prognostic studies, the presence of a CCTA-defined high-risk plaque was associated with future cardiovascular events, but the improvement in prognostication compared with conventional clinical risk stratification was surprisingly low [34]. Therefore, current European and American guidelines do not translate the evidence on the association between vulnerable plaques and future cardiac events into recommendations for the guidance of specific preventive actions. The limitation lies in the lesion-focused approach: while features such as thin fibrous caps, large lipid cores, and macrophage infiltration are associated with adverse outcomes, they are rare, and their individual prognostic value remains modest [35].

The PROSPECT study enrolled 697 post-ACS patients who underwent three-vessel IVUS and VH-IVUS imaging after percutaneous coronary intervention (PCI), “vulnerable plaque” was defined by plaque burden ≥ 70%, MLA ≤ 4.0 mm^2^, and VH-defined TCFA. Over 3.4 years, only 11.6% of MACE were linked to non-culprit lesion progression, with a positive predictive value (PPV) of 18.2% for plaques meeting all criteria. Notably, cardiovascular death, cardiac arrest, or MI occurred in only 4.9% of patients [36].

PROSPECT II imaged 3629 non-culprit lesions in 898 patients with recent myocardial infarction (MI) using NIRS-IVUS. The identified high-risk features included lipid-rich plaques (lipid core burden index, LCBI > 400), plaque burden ≥ 70%, and MLA ≤ 4.0 mm^2^. At the patient level, 34% had at least one lesion with both high lipid content and large burden, yet the 4-year MACE rate among them was only 13%; although lipid-rich and large-burden plaques were statistically associated with increased risk, the PPV at the lesion level was low (7%) [37].

In the Lipid-Rich Plaque (LRP) study (n = 1271), patients were followed for 2.1 years after NIRS-IVUS imaging. Despite high LCBI (≥400) in 39% of patients, the rate of non-culprit lesion-related MACE was only 9%. Many high-risk plaques remained clinically silent, resulting in low specificity and modest positive predictive value [2].

The CLIMA study analysed 1776 plaques in 1003 patients with OCT of the proximal LAD. Only 3.6% of plaques met all four high-risk criteria (thin fibrous cap < 75 μm, large lipid arc, macrophage infiltration, and MLA < 3.5 mm^2^), with a 12-month MACE rate of 18.9% in this subgroup versus 3.0% in others (HR 7.54). However, this high-risk subset represented just 1.8% of patients, which substantially limits the predictive value [25].

PROSPECT and PROSPECT II focused on post-ACS patients, LRP mainly enrolled patients post-MI, and CLIMA included a mixed population with approximately half having ACS and the rest stable coronary disease. Therefore, all studies were conducted in a secondary prevention context. Even in these high-risk cohorts, the PPV of vulnerable plaque features remained modest, highlighting that lesion-focused imaging alone is insufficient to accurately stratify future risk [34].

## 5. Clinical Settings and Limitations of Current Imaging Strategies

While numerous imaging modalities can identify features of plaque vulnerability, their clinical utility is context-dependent and influenced by patient presentation, procedural indications, and resource availability.

### 5.1. Scenario-Based Use in Current Practice

- *ACS and PCI guidance:* OCT and IVUS constitute the principal intraprocedural tools. OCT enables high-resolution visualisation of fibrous cap integrity, rupture versus erosion, thrombus, and stent–vessel interactions, but its shallow penetration depth, reliance on transient blood clearance with contrast, and inability to quantify overall plaque burden restrict broader applicability [21,38,39]. IVUS, including its virtual histology variant, remains indispensable for assessing plaque burden, vessel remodelling, stent expansion, and left main or complex coronary anatomy. Yet, the limited axial resolution precludes accurate evaluation of cap thickness or macrophage infiltration, and procedural time and operator variability remain non-trivial concerns [40]. Moreover, in ACS, intravascular imaging is increasingly used to analyse non-culprit plaques to identify features of vulnerability [41]. Frequently, multiple vulnerable plaques coexist, creating uncertainty regarding lesion selection for preventive stenting and raising concerns about overtreatment. In addition, OCT or IVUS may assist in identifying the culprit lesion when angiography is inconclusive, but performing pullbacks in multiple vessels or segments is time-consuming and often impractical in the acute setting [42]. Finally, in patients presenting with acute chest pain without overt ST-Elevation or clear evidence of obstructive CAD, CCTA focused on lesion-level assessment and integrated with Computed Tomography-derived Fractional Flow Reserve (CT-FFR) may increase the negative predictive value of the examination and inform early invasive management when appropriate [43]. This approach can help identify haemodynamically relevant or high-risk plaques while safely ruling out obstructive disease [43].
- *Stable chest pain and chronic coronary syndromes:* CCTA is now established as a first-line diagnostic test in patients with a low-to-intermediate (5–50%) pre-test likelihood of obstructive CAD [27]. Beyond excluding obstructive disease, CCTA enables quantification of total plaque burden, low-attenuation components, remodelling indices, and high-risk morphologies such as the napkin-ring sign [44]. Automated plaque quantification is increasingly favoured over segment-focused visual interpretation, as total burden appears to outperform isolated high-risk features in predicting events [34]. Notably, AI-assisted coronary plaque analysis (AI-CPA) has recently received FDA clearance for automated quantification of atherosclerotic burden on CCTA, marking a potential shift from lesion-based assessment to comprehensive risk phenotyping. This approach may prove particularly relevant in patients with low or zero calcium scores, in whom traditional CAC assessment underestimates non-calcific disease [45]. The rapidly expanding incorporation of CT- FFR and perivascular fat attenuation index (FAI) offers an emerging functional and inflammatory dimension to coronary phenotyping [46,47]. Nonetheless, radiation exposure, susceptibility to blooming and motion artefacts, the variable availability of advanced post-processing platforms, and unresolved questions of cost-effectiveness and standardisation limit universal adoption [8,48].
- *Myocardial infarction with Non-Obstructive Coronary Arteries (MINOCA) and uncertain culprit scenarios:* In patients with MINOCA, or when a culprit lesion cannot be identified despite angiography and intravascular imaging, plaque assessment with CMR may provide a comprehensive alternative [49]. When combined with myocardial tissue characterisation, a single examination could clarify both the vascular substrate and the downstream myocardial consequences [49]. However, the absence of dedicated plaque imaging sequences and the current lack of widespread technology limit routine implementation.
- *High-risk or research-based clinical cohorts:* NIRS-IVUS, PET, and advanced CMR imaging provide insights into the biological activity of atherosclerotic disease that remain inaccessible to conventional modalities. NIRS-IVUS enables direct lipid core quantification and has demonstrated prognostic relevance in prospective natural history cohorts [50]. However, its predictive value at the lesion level remains modest and the technology is largely confined to specialised centres due to cost and limited accessibility [50]. PET tracers targeting inflammation and CMR sequences capable of detecting intraplaque haemorrhage, neovascularisation, or fibrosis have expanded the conceptual framework of vulnerability, but their clinical applicability is constrained by spatial resolution, scan time, patient selection, and reimbursement barriers [33,51].

### 5.2. Future Perspectives and Emerging Clinical Applications

Early data suggest that emerging imaging strategies may soon influence therapeutic decision-making, and vulnerability-directed imaging may evolve from risk description to therapeutic guidance. AI-assisted IVUS/OCT has been tested during ACS to identify non-culprit plaques with cap thinning or inflammatory features, prompting intensified medical therapy rather than routine stenting [52]. CCTA-derived vulnerability markers (e.g., low-attenuation burden, FAI, rapid plaque progression) are being used to select patients for PCSK9 inhibitors, colchicine, or dual-pathway anti-thrombotic therapy, even in the absence of obstructive lesions [53]. NIRS-IVUS has been applied to identify high-lipid burden plaques for potential preventive intervention, as explored in PROSPECT ABSORB [54]. Biomechanical parameters such as radial wall strain are under investigation to prioritise treatment of angiographically intermediate lesions that appear stable but demonstrate mechanical instability, as has been observed, for example, in patients with acute myocardial infarction following complete revascularisation [55].

## 6. Phenotyping the Biochemical Environment of Vulnerability

### 6.1. Lipid-Related Risk Signatures

While elevated low-density lipoprotein (LDL) cholesterol has long represented the cornerstone of atherogenic risk, additional lipid parameters have emerged as potential modulators of plaque biology [56]. Lipoprotein(a) [Lp(a)], a genetically determined LDL-like particle containing apolipoprotein(a), exerts pro-atherogenic, pro-inflammatory, and pro-thrombotic effects and is minimally affected by lifestyle or diet [56]. In patients with established coronary artery disease, elevated Lp(a) levels (>70 mg/dL) have been linked to accelerated progression of low-attenuation plaque on CCTA, a surrogate of necrotic core and high-risk morphology [57]. More recently, CCTA-based studies have shown that high Lp(a) correlates with increased total plaque volume, abnormal fat attenuation index, and lower CT-FFR values, suggesting interplay between lipid burden, inflammatory activation, and impairment in coronary physiology [58].

**Figure 3:**
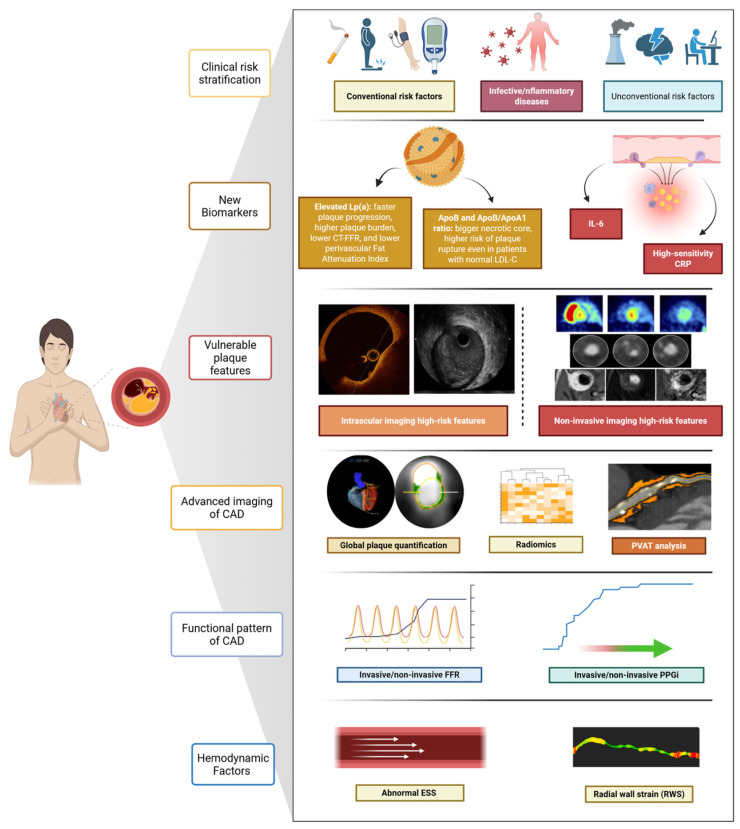
**Multidimensional assessment of coronary vulnerability.** Comprehensive evaluation of coronary risk integrating clinical risk factors, circulating biomarkers, and vulnerable plaque features with advanced imaging and functional assessments.

Apolipoprotein B (ApoB) and the ApoB/Apolipoprotein A1 (ApoA1) ratio have also been investigated as correlates of adverse plaque characteristics [59]. Data from the AMORIS study demonstrated that ApoB and the ApoB/ApoA1 ratio improve risk prediction for fatal myocardial infarction beyond LDL-C in large populations [59]. In more recent intravascular imaging cohorts, higher ApoB levels have been associated with greater necrotic core volume and longer total plaque length, particularly in patients with stable coronary artery disease undergoing VH-IVUS [60]. These findings support the concept that individuals with elevated ApoB or an unfavourable ApoB/ApoA1 ratio tend to exhibit more advanced or high-risk plaque characteristics. However, unlike Lp(a)—which is independent of LDL levels and not modified by statin therapy—the ApoB/ApoA1 ratio has not yet shown clear added value in primary prevention beyond current lipid-based risk assessment frameworks, Figure 3.

### 6.2. Phenotyping the High-Risk Inflammatory Profile

Data from the proof-of-concept CANTOS (Canakinumab Anti-inflammatory Thrombosis Outcome Study) trial have clearly identified inflammation as one of the critical biological processes leading to coronary events [61]. Systemic inflammation, driven by metabolic conditions and risk factors such as obesity, chronic kidney disease, rheumatoid arthritis, and smoking, accelerates plaque progression and increases clinical event rates [53]. Traditional markers of systemic inflammation, such as high-sensitivity C-reactive protein (hsCRP), appear to have limited predictive power for individual risk due to their lack of specificity and direct causal association with atherosclerosis (hsCRP levels are influenced by age, sex, infections, autoimmune disorders, and cardiometabolic comorbidities, which limits their interpretability at the patient level). However, in patients receiving more intensive lipid-lowering therapy, hsCRP was a stronger predictor of future cardiovascular events than LDL alone [53]. Inflammatory markers such as interleukin-6 (IL-6), fibrinogen, and homocysteine have also been associated with high-risk plaque features detected by IVUS and OCT, including TCFA and large plaque burden. However, these associations are not lesion-specific and largely reflect systemic inflammatory activity rather than plaque vulnerability. Although elevated IL-6 levels have been associated with a higher risk of cardiovascular events independently of traditional risk factors, no circulating biomarker has yet shown sufficient specificity or discriminative power to identify which lesions are truly at imminent risk, and systemic inflammatory markers remain prognostic only at the population level [53,62].

In the COLCOT (Colchicine Cardiovascular Outcomes Trial) study, low-dose colchicine effectively prevented MACE in patients who experienced a recent MI compared with placebo [63]. As a result, the FDA approved using low-dose colchicine, and the European Society of Cardiology guidelines recommended considering colchicine as an option to standard care to prevent recurrent cardiovascular events in patients with acute coronary syndromes [29]. Notably, the results of the COCOMO-ACS trial (COlchicine for COronary Plaque MOdification in Acute Coronary Syndrome) found that OCT did not detect a significant effect of colchicine on minimum fibrous cap thickness, maximum lipid arc in non-culprit imaged segments, or lipid-rich plaques, despite the evident benefit of anti-inflammatory therapy on patients’ residual risk of events [64].

This dissociation between systemic biomarker modulation and plaque morphology underscores the limitations of inflammation markers as standalone tools and reinforces the need for integrative approaches that incorporate imaging, biology, and patient-specific risk profiling. Again, these data highlight the limitation of a “plaque-centred” approach to patient risk stratification, Figure 3.

## 7. Integrating New Imaging Strategies to Define the “Vulnerable” Phenotype

### 7.1. AI

AI has rapidly gained attention as a complementary tool in cardiovascular imaging, enhancing the detection, quantification, and prognostication of vulnerable plaques: its application spans both non-invasive (CCTA) and invasive imaging (IVUS, OCT) [65,66]. In IVUS, AI-based image interpretation is challenged by limited spatial resolution, leading to the use of per-frame or circumferential plaque segmentation. Despite this, deep learning models referencing OCT have shown promising results in identifying TCFA (AUC 0.84–0.91), while Dice similarity coefficients for detecting calcified and attenuated plaques reached 0.79 and 0.74, respectively [67]. In OCT, A-line-based classification rearranges cross-sectional data to analyse plaques circumferentially, enabling fibrous cap detection even in lipid-rich plaques that obscure the external elastic membrane. Pixel-based deep learning models have demonstrated good diagnostic accuracy (~87%) and Dice coefficients around 0.76. These tools effectively identify lipidic and calcified plaques (sensitivity/specificity > 85%) and detect TCFA with AUCs up to 0.96, significantly reducing interpretation time [68]. The Exploring the Mechanism of Plaque Rupture in Acute Coronary Syndrome Using Coronary Computed Tomography Angiography and Computational Fluid Dynamics II (EMERALD-II) study applied AI-enabled analysis of CCTA and haemodynamics in 351 ACS patients, demonstrating superior event prediction when AI-derived features (FFR, plaque burden, low-attenuation volume, perfusion metrics) were added to conventional risk models (AUC 0.84 vs. 0.78; *p* < 0.001) [69]. Similarly, in ISCHEMIA, atherosclerosis imaging quantitative computed tomography analysis of 3711 CCTAs showed total plaque volume to be the strongest predictor of adverse outcomes, improving model performance at six months, two years, and four years [70], Figure 3.

### 7.2. New CCTA-Derived Analyses to Refine Disease Phenotype

Advances in CCTA now permit automated quantification of total coronary plaque burden and its components: calcific, fibrotic, lipidic, and low-attenuation plaques (<30 HU, indicating necrotic cores) [71]. These quantitative plaque burden measurements appear robust, with good observer variability, scan–rescan reproducibility, and close correlation with intravascular ultrasound assessments, and, as shown recently, can be derived within seconds using deep learning [71]. In the SCOT-HEART trial, low-attenuation plaque burden, assessed via semiautomated software, was the strongest predictor of MI at 4.7 years, surpassing traditional high-risk features [72]. Quantifying total and component-specific plaque burden may overcome the limitations inherent to single high-risk features, recognising that the cumulative atherosclerotic load better reflects overall risk [73]. An emerging imaging biomarker in CCTA analysis is the perivascular FAI, a surrogate measure of peri-coronary fat inflammatory activity. FAI captures spatial gradients in attenuation, potentially reflecting dynamic changes driven by inflammation. In the CRISP-CT study, FAI ≥ –70 HU was associated with a five- to nine-fold increased risk of cardiac death, independent of plaque burden [46]. The multicentre, longitudinal Oxford Risk Factors and Non-invasive imaging (ORFAN) study analysed over 40,000 consecutive patients undergoing CCTA, showing that elevated FAI scores in all three coronary arteries markedly increased the risk of MACE [47]. The AI-derived risk classification model correlated with outcomes and remained predictive independent of plaque burden or stenosis severity [47]. Despite its specificity, FAI is not yet integrated into clinical workflows. No validated cut-offs exist for therapeutic decision-making, and whether FAI will evolve from a prognostic marker to a decision-making tool will depend on prospective validation and integration with plaque burden, CT-FFR, and systemic biomarkers. Furthermore, recent studies combine AI-based analysis with functional metrics in CT-FFR: a retrospective study by Von Knebel Doeberitz et al. reported that combining CCTA-derived plaque features with ML-based CT-FFR significantly improved prediction of MACE (AUC 0.94 vs. 0.60 for stenosis alone) [74]. These data suggest that a combined anatomical, functional, and inflammatory assessment of coronary disease—now technically feasible using CCTA—may offer incremental value in risk stratification. However, no current evidence supports its integration into routine decision-making. As proposed by Sato and colleagues, this approach could help refine the definition of “significant” disease and address limitations of past trials comparing revascularisation with optimal medical therapy, but such assumptions still require confirmation in outcome-driven studies [34,75]. The non-invasive nature of CCTA has also prompted interest in serial imaging for selected high-risk patients. Monitoring temporal changes in plaque burden, CT-FFR, or FAI could theoretically improve risk assessment, but concerns regarding radiation exposure, cost-effectiveness, patient selection, and standardisation currently restrict this strategy to investigational settings [76]. Recently, CONFIRM II was designed: the multicentre, international trial will examine the relationships between CCTA-derived coronary phenotypes, serum biomarkers, clinical presentation, and outcomes. Automated software will be employed to quantify plaque, stenosis, and cardiac structures, facilitating standardised assessment and follow-up over time [77], Figure 3. Its results will be essential to determine whether CCTA, when combined with AI, biomechanical indices, or biological markers, can progress beyond descriptive characterisation and inform therapeutic strategies.

### 7.3. Integrating Haemodynamic and Biomechanical Factors to Identify Vulnerable Phenotypes

Haemodynamic and biomechanical forces critically influence atherosclerotic plaque initiation, progression, and destabilisation; plaque vulnerability results from the interplay between composition and mechanical forces, which are modulated by age and risk factors. Assessing vessel biomechanics provides additional insights into coronary pathophysiology and complements morphological plaque evaluation [78]. Repetitive circumferential wall stress and radial strain are key mechanical factors in fibrous cap thinning and rupture. Coronary strain reflects arterial wall deformation due to circumferential stress (pulsatile pressure) and is directly influenced by tissue stiffness and plaque composition. Vulnerable plaques, rich in lipids or macrophages, typically show high strain values, whereas stabilising components such as fibrous tissue or calcium correspond to lower strain values. Thus, areas of high strain may identify plaques with a greater likelihood of causing ACS. Superficial wall strain and stress (SWS) encompass multiple vectors, one of which is radial wall strain (RWS)—a directional component increasingly studied as a standalone indicator of mechanical vulnerability [78,79,80], Figure 3. Historically, coronary strain was measured using IVUS-based elastography and palpography. These modalities showed that high-strain regions aligned with vulnerable plaques, but clinical applicability remained limited due to technical complexity and lack of standardisation [81]. Vessel geometry and plaque location also shape flow dynamics and endothelial shear stress (ESS)—the tangential frictional force exerted by blood flow on the endothelial surface [78,82]. Both high and low ESS are important in plaque evolution: low ESS promotes smooth muscle migration, intimal thickening, and fibroatheroma development, whereas high ESS enhances proteolytic activity through metalloproteinase activation, thinning the fibrous cap [78]. The PREDICTION study (n = 506 ACS) showed that combining low ESS (<1.0 Pa) with high plaque burden (≥58%) predicted progressive plaque growth and vessel narrowing, with a 41% positive predictive value for clinical events. Using angiography, IVUS, and 3D flow mapping, this study highlighted the importance of haemodynamic–biological interactions [83]. Similarly, a sub-analysis of 2018 FAME II involving 441 chronic coronary syndrome patients with positive FFR demonstrated that angiography-derived high ESS in proximal segments predicted MI over a three-year period when compared to other vessel wall regions. Okamoto et al. further confirmed the association between high ESS and the presence TCFA in 85 patients, identifying a cut-off of 6.79 Pa as predictive in proximal obstructive segments [84]. Radial wall strain (RWS) has emerged as a non-invasive, angiography-derived biomechanical index to assess vulnerability. Enabled by AI, RWS quantifies radial deformation (%) from single-view angiography, avoiding the need for intravascular imaging. Its computational simplicity, broad applicability, and avoidance of procedural burden make it an attractive clinical tool [79,81]. In validation studies, maximum RWS positively correlated with OCT-derived lipid burden and lipid-to-cap ratio, and negatively with fibrous cap thickness, Table 1. In a post hoc analysis of the FAVOR III China study (824 vessels, 751 patients), an RWS > 12% was a strong independent predictor of one-year vessel-oriented composite endpoints (adjusted HR: 4.44; 95% CI: 2.43–8.14; *p* < 0.001) [79,85], Table 1. Building on these findings, the ongoing FAVOR V AMI trial (NCT05669222)—a multicentre, randomised, sham-controlled study—is investigating the clinical utility of RWS in over 5000 ST-Elevation Myocardial Infarction (STEMI) patients with multivessel disease. Following successful treatment of culprit artery, patients are randomised to μQFR- and RWS-guided revascularisation versus standard care for non-culprit lesions. The trial tests the hypothesis that combining physiological and biomechanical plaque assessment improves outcomes by guiding revascularisation more precisely [86,87,88].

## 8. Unmet Needs and Gaps in Evidence

Despite major technical advances, no single modality has yet demonstrated sufficient predictive accuracy to guide prophylactic or lesion-targeted interventions. The integration of multimodality imaging, systemic biomarkers, AI-derived analytics, and biomechanical indices may enhance patient-level risk stratification; however, cost-effectiveness, workflow compatibility, and validation in prospective randomised trials remain unresolved. A fully integrated CCTA platform capable of combining anatomical assessment (atherosclerotic burden and composition), functional significance (CT-FFR), and biological vulnerability (e.g., perivascular FAI or alternative inflammatory markers) represents a logical and increasingly feasible direction for development. Nevertheless, such an approach is still investigational: standardisation of acquisition protocols, harmonisation of post-processing software, reduction in radiation exposure, and prospective outcome data are essential prerequisites before it can influence clinical decision-making.

In addition, several unresolved challenges restrict clinical implementation. No validated thresholds currently exist for FAI, CT-FFR, or biomechanical indices to guide therapy, and uncertainty persists about which patients would benefit from advanced phenotyping, whether serial imaging is justified, and how multimodal findings should influence management decisions. The prognostic value of combining anatomy, function, inflammation, and mechanics remains largely observational, and it is unknown whether vulnerability-guided strategies improve outcomes compared with current risk-based approaches. The concept of a “one-stop” CCTA-based phenotype is therefore promising but not yet practice-changing, and prospective validation studies will be essential to determine whether this model can evolve from a prognostic concept to an actionable clinical strategy.

## 9. Conclusions

Despite significant advances in plaque imaging and characterisation, the plaque-centric approach has shown limited predictive value for future events. Emerging evidence supports a shift toward an integrated assessment of coronary vulnerability, encompassing anatomical, functional, inflammatory, and biomechanical dimensions. This evolving concept of a “vulnerable phenotype” moves beyond single imaging high-risk features to embrace a patient-level risk profile. The integration of advanced imaging, AI, and biological markers may enable more precise risk stratification and guide individualised preventive strategies, marking a pivotal evolution in the management of CAD.

## Figures and Tables

**Table 1:** Overview of RWS validation studies.

Author, Journal	Study Design	FU Duration	N° Patients	Rate of Events	Prognostic Value
Zhiqing Wang et al. Journal of Geriatric Cardiology [86]	Retrospective analysis of a clinical registry	Median follow-up 16.8 months	603	Angiographic progression occurred in 49 lesions in 49 patients	RWSmax > 12.6% was independently associated with an increased risk of lesion progression (adjusted HR = 6.82)
Chenguang Li et al. JACC: Cardiovascular Interventions [87]	Retrospective analysis of a clinical registry	2 years	44 (matched with 132 controls)	44 patients with lesion-related AMI	RWSmax > 12% was found independently associated with subsequent AMI events (RR = 7.25)
Shengxian Tu et al. JACC [85]	Post hoc analysis of the RCT FAVOR III China	1 year	751 patients	VOCE occurred in 46 out of 824 vessels	RWSmax > 12% was an independent predictor of 1-year VOCE in deferred non-flow limiting vessels (adjusted HR = 4.44)
Huihong Hong et al. EuroIntervention [79]	Post hoc analysis of 124 vessels with OCT assessment	NA	114 patients	NA	RWS correlated positively with lipid cap ratio (r = 0.584; *p* < 0.001) lipidic plaque burden (r = 0.411; *p* < 0.001), and negatively with fibrous cap thickness (r = −0.439; *p* < 0.001). RWSmax > 12% predictor for a lipid cap ratio > 0.33 (area under the curve [AUC] = 0.86, 95% confidence interval [CI]: 0.78–0.91; *p* < 0.001) and TCFA (AUC = 0.72, 95% CI: 0.63–0.80; *p* < 0.001)
Jiayue Huang et al. JSCAI [88]	In silico model methodology comparison study RWSAngio vs. RWS OCT	NA	36 patients (45 lesions)	NA	RWSAngio showed good correlation and agreement with RWSOCT (r = 0.91; *p* < 0.001). RWSAngio in atherosclerotic segments was significantly higher than that in healthy segments (12.6% [11.0, 16.0] vs. 4.5% [2.9, 5.5], *p* < 0.001).

## Data Availability

No new data were created or analyzed in this study.

## Abbreviations

The following abbreviations are used in this manuscript:

ACS	Acute Coronary Syndrome
AI	Artificial Intelligence
AI-CPA	AI-assisted Coronary Plaque Analysis
ApoA1	Apolipoprotein A1
ApoB	Apolipoprotein B
AUC	Area Under the Curve
CAD	Coronary Artery Disease
CCTA	Coronary Computed Tomography Angiography
CMR	Cardiac Magnetic Resonance
CT-FFR	Computed Tomography-Derived Fractional Flow Reserve
ESS	Endothelial Shear Stress
FAI	Fat Attenuation Index
FDA	Food and Drug Administration
FFR	Fractional Flow Reserve
hsCRP	High-Sensitivity C-Reactive Protein
IL-6	Interleukin-6
IVUS	Intravascular Ultrasound
LAD	Left Anterior Descending artery
LCBI	Lipid Core Burden Index
LDL	Low-Density Lipoprotein
Lp(a)	Lipoprotein(a)
MACE	Major Adverse Cardiac Events
MI	Myocardial Infarction
MINOCA	Myocardial Infarction with Non-Obstructive Coronary Arteries
MLA	Minimal Lumen Area
μQFR	Murray Law-Based Quantitative Flow Ratio
NIRS	Near-Infrared Spectroscopy
OCT	Optical Coherence Tomography
PCCT	Photon-Counting Computed Tomography
PCI	Percutaneous Coronary Intervention
PET	Positron Emission Tomography
PPV	Positive Predictive Value
RWS	Radial Wall Strain
STEMI	ST-Elevation Myocardial Infarction
SWS	Superficial Wall Strain/Stress
TCFA	Thin-Cap Fibroatheroma
VH-IVUS	Virtual Histology Intravascular Ultrasound
VOCE	Vessel-Oriented Composite Endpoint
